# Postpartum Prolapsed Leiomyoma with Uterine Inversion Managed by Vaginal Hysterectomy

**DOI:** 10.1155/2014/435101

**Published:** 2014-10-14

**Authors:** Kelly L. Pieh-Holder, Heidi Bell, Tana Hall, James E. DeVente

**Affiliations:** Department of Obstetrics and Gynecology, Brody School of Medicine, East Carolina University, Greenville, NC 27834, USA

## Abstract

*Background*. Uterine inversion is a rare, but life threatening, obstetrical emergency which occurs when the uterine fundus collapses into the endometrial cavity. Various conservative and surgical therapies have been outlined in the literature for the management of uterine inversions. *Case*. We present a case of a chronic, recurrent uterine inversion, which was diagnosed following spontaneous vaginal delivery and recurred seven weeks later. The uterine inversion was likely due to a leiomyoma. This late-presenting, chronic, recurring uterine inversion was treated with a vaginal hysterectomy. *Conclusion*. Uterine inversions can occur in both acute and chronic phases. Persistent vaginal bleeding with the appearance of a prolapsing fibroid should prompt further investigation for uterine inversion and may require surgical therapy. A vaginal hysterectomy may be an appropriate management option in select populations and may be considered in women who do not desire to maintain reproductive function.

## 1. Introduction

Uterine inversion is a rare, but life threatening, obstetrical emergency which occurs when the uterine fundus collapses into the endometrial cavity. Uterine inversion may be classified as puerperal (obstetric) or nonpuerperal (gynecologic), may occur in varying degrees, from fundal dimpling to prolapse of the uterus and cervix, and can be seen in acute and chronic forms. Various conservative and surgical therapies have been outlined in the literature for the management of uterine inversions. We present a case of a postpartum prolapsed leiomyoma associated with chronic, recurring uterine inversion treated with a vaginal hysterectomy.

## 2. Case Presentation

A forty-year-old G3, now P2012, underwent a spontaneous vaginal birth after cesarean section presented to labor and delivery in active labor at term. The patient delivered a viable female infant weighing 3757 grams with APGARs of 8 and 9 at 1 minute and 5 minutes, respectively. Following spontaneous delivery of the placenta, the fundus was noted to be slightly prolapsed through the internal os. A fourth degree laceration occurred at the time of delivery due to the emergent maneuvers utilized to replace the uterus. Brisk vaginal bleeding was noted and 500 cc of blood clot was removed the uterus. Given the patient's inability to tolerate the exam, 1000 mcg of cytotec was placed rectally and the patient was moved to the operating room where general anesthesia was administered. Manual exploration of the uterus was again performed and an additional 1000 cc of blood clot was removed and an anterior fibroid with partial prolapse of the uterus was noted. The fundus was manually replaced superiorly and attention was then turned to the 4th degree laceration which was repaired in standard fashion without complication. Following the 4th degree repair, the uterus was noted to be atonic again and uterine massage and manipulation elicited an additional 500 cc of blood clot. The patient was given two doses of hemabate 250 mcg and the uterus was noted to be firm and hemostasis was confirmed. Total blood loss at the time of delivery was estimated to be 4.5 liters and the patient received 8 units of packed red blood cells (pRBCs) and 4 units of fresh frozen plasma. The postpartum course was uncomplicated and she was discharged home on postpartum day three.

At seven weeks postpartum the patient presented to the local health department with complaints of persistent vaginal bleeding since delivery. Upon speculum exam, she was noted to have a 5 cm prolapsed vaginal fibroid and was referred to the local tertiary center. Initial gynecology exam confirmed a prolapsing vaginal fibroid and she was consented for a vaginal myomectomy, possible hysterectomy and blood.

**Figure 1:**
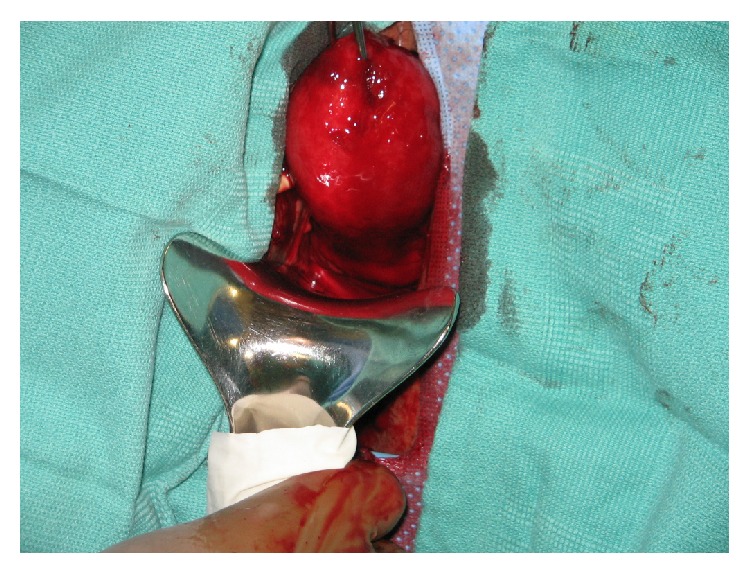
Uterine inversion.

**Figure 2:**
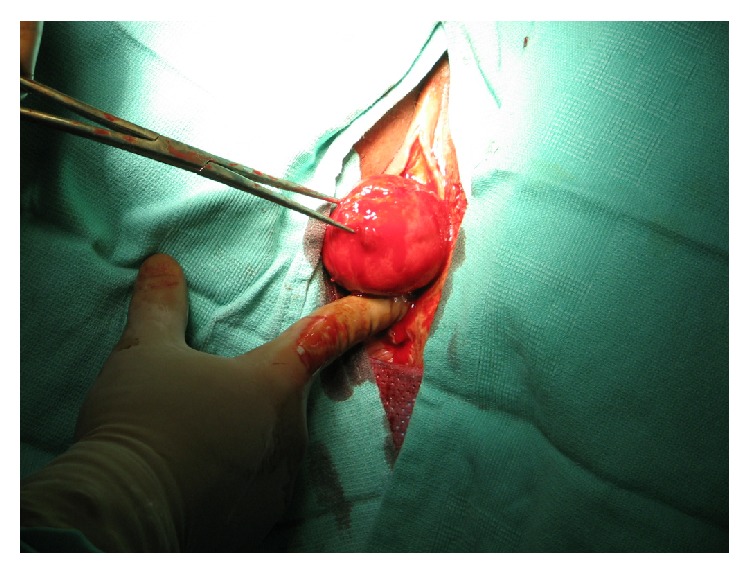
Uterine inversion.

Examination under general anesthesia was performed in which rectal and bimanual examination revealed the presence of a uterine inversion (Figures 1 and 2). Vaginal attempts to evert the uterus were unsuccessful due to a tight ring of tissue noted at the cervix. The anterior compartment of the peritoneal cavity was then entered bluntly, secondary to necrotic tissue. A compression defect was noted on the posterior wall of the bladder likely secondary to the prolonged mass effect of the uterus within the vagina. Bilateral ureteral stents were then placed due to the concern for the location of the ureters. A vaginal hysterectomy was then performed. Due to complete uterine prolapse, the uterosacral ligament, cardinal ligament, and the uterine artery pedicle were clamped, incised, and suture ligated in a single pass. Good hemostasis was confirmed and closure of the vaginal cuff was then performed. A cystoscopy was performed at the end of the case which confirmed patent and functioning ureters and an intact bladder. Estimated blood loss was 600 cc and the patient received 2 liters of crystalloids, 1 unit pRBCs with pre- and postoperative hematocrit of 32.1 and 26.7, respectively. Pathology of the uterus showed degenerating endometrium with stromal bleeding and intramural leiomyoma with severe ischemic changes. The patient was discharged on postoperative day one in stable condition and was doing well at her six-week postoperative visit.

## 3. Discussion

The majority of uterine inversions occur during the puerperal period with the incidence being anywhere from 1 in 2148 to 1 in 6407 vaginal births [1, 2]. In a 20-year literature review by Dali et al., 241 cases of uterine inversion were identified in the literature. Two hundred twenty-nine cases of uterine inversion occurred during the puerperal phase while only twelve occurred during the nonpuerperal phase. Among the puerperal uterine inversions, 83.4% were acute, 2.62% were subacute, and 13.9% were chronic [3].

Uterine inversions may be classified by severity. An incomplete uterine inversion occurs when the fundus lies within the endometrial cavity, a complete uterine inversion occurs when the fundus protrudes through the external cervical os, and a prolapsed inversion occurs when the fundus extends into and through the introitus. Uterine inversions may also be classified as acute, occurring within 24 hours of delivery, subacute, occurring more than 24 hours after delivery, or chronic, occurring more than one month postpartum. The length of time to diagnosis of postpartum chronic uterine inversion has been noted to occur anywhere from four to fourteen weeks [4–7], with our case being rediagnosed at seven weeks postpartum.

Risk factors for a uterine inversion may include fetal macrosomia, rapid labor and delivery, short umbilical cord, use of uterine relaxants, uterine anomalies, manual removal of the placenta, placenta accreta, fundal pressure prior to placental separation, or leiomyomas. Puerperal inversions typically occur following a spontaneous vaginal delivery, but may also occur through the hysterotomy incision at the time of cesarean delivery [8]. Nonpuerperal uterine inversions are frequently associated with uterine pathology such as leiomyomas, endometrial polyps, or carcinomas [9, 10].

Diagnosis of a uterine inversion has been shown to be quite difficult. On physical exam, a uterine inversion typically reveals a fundal notch, although the most common presentation is clinical shock, out of proportion with a postpartum hemorrhage. This clinical shock and frequently associated bradycardia is thought to be due to the parasympathetic effect of the traction on the supporting uterine ligaments [11]. However, several authors have speculated that this classical description of shock, out of proportion to blood loss, may more likely represent underestimation of blood loss [2, 12] and should be viewed with caution, as this can be misleading in regard to emergent management. Chronic uterine inversions typically present with anemia due to irregular bleeding, vaginal discharge, pelvic pain, and a protruding mass in the vagina. In addition to physical exam, diagnosis can be aided by the use of various imaging modalities with MRI being more effective than computed tomography or ultrasonography in making the diagnosis [6, 13–15].

Successful management depends on prompt recognition, correction of the inversion, and immediate treatment of hemorrhagic shock. Resuscitation should begin immediately while attempts are made to manually replace the uterus, leaving the placenta in place until the uterus has been replaced [1]. Management involves the discontinuation of uterotonic drugs. Uterine inversion may be easily mistaken for an extruding leiomyoma, but it must not be overlooked as a possible cause of hemorrhage during the immediate or delayed postpartum period. In order to prevent complete reinversion of the uterus, it is important to consider the diagnosis of uterine inversion prior to the utilization of uterotonics. Uterine relaxation is needed for the complete replacement of the uterus. If contraction of the cervix has occurred, magnesium sulfate, nitroglycerin, or beta-adrenergic agents such as terbutaline may be used if necessary to relax the uterus. Anesthetic agents such as halothane or enflurane may also be used as uterine relaxants [16]. Management following successful replacement of the uterus often includes uterotonics such as oxytocin, hemabate, cytotec, or methergine.

If manual reduction is unsuccessful, alternate conservative or surgical approaches may be attempted with chronic cases being more likely to require surgical correction [13]. Conservative management options previously described include hydrostatic pressure to reinvert the uterus [17, 18], hydrostatic balloon placement [19], and placement of the SOS Bakri balloon for chronic recurrent uterine inversion [20]. Surgical therapy has also been described in the literature for over one hundred years. In 1901, Dr. Haultain described The Haultain procedure, which can be performed either vaginally or abdominally. An incision is made on the posterior aspect of the cervicoisthmic constriction to increase the diameter, and the uterine fundus is repositioned [21]. The Huntington procedure requires a laparotomy to locate the fundus. Clamps are placed on the round ligaments and gentle upward traction is applied [22]. A modified Haultain approach via combination of an abdominal and transvaginal approach has also been described [4]. Spinelli [23] and Kustner procedures [24] describe a transvaginal approach of releasing the posterior cervical constriction ring through the posterior cul de sac. Additional options, such as abdominal placement and cerclage [25] and transvaginal tumor resection with reinversion of the uterus, have also been described [6]. Lastly, a laparoscopic approach [26] and combined laparoscopic and vaginal approach have also been reported [27].

## 4. Conclusion

This case report presents a chronic, recurrent uterine inversion managed with a vaginal hysterectomy. Several factors may have played a role in this case. Two unique factors which played a role in this case were the presence of a large, mucosal uterine fibroid and the inversion of the uterus in the immediate puerperal. This case also highlights the possibility that a uterine inversion can easily be mistaken for a leiomyoma. In patients who do not desire to preserve future fertility, a vaginal hysterectomy may be an appropriate management option for recurrent uterine inversion or uterine inversion due to prolapsing leiomyoma.

## References

[1] Platt L. D., Druzin M. L. (1981). Acute puerperal inversion of the uterus. *American Journal of Obstetrics and Gynecology*.

[2] Shah-Hosseini R., Evrard J. R. (1989). Puerperal uterine inversion. *Obstetrics and Gynecology*.

[3] Dali S. M., Rajbhandari S., Shrestha S. (1997). Puerperal inversion of the uterus in Nepal: case reports and review of literature. *Journal of Obstetrics and Gynaecology Research*.

[4] Livingston S. L., Booker C., Kramer P., Dodson W. C. (2007). Chronic uterine inversion at 14 weeks postpartum. *Obstetrics & Gynecology*.

[5] Sheikh H. H. (1998). Uterine leiomyoma as a rare cause of acute abdomen and intestinal gangrene. *American Journal of Obstetrics and Gynecology*.

[6] Shirota K., Ota T., Tsujioka H., Miyamoto S. (2011). Uterine inversion due to a leiomyoma on postpartum day 41: a case report. *Journal of Obstetrics and Gynaecology Research*.

[7] Antonelli E., Irion O., Tolck P., Morales M. (2006). Subacute uterine inversion: description of a novel replacement technique using the obstetric ventouse. *BJOG*.

[8] Marshall N. B., Catling S. (2010). Cardiac arrest due to uterine inversion during caesarean section. *International Journal of Obstetric Anesthesia*.

[9] Gomez-Lobo V., Burch W., Khanna P. C. (2007). Nonpuerperal uterine inversion associated with an immature teratoma of the uterus in an adolescent. *Obstetrics and Gynecology*.

[10] Rocconi R., Huh W. K., Chiang S. (2003). Postmenopausal uterine inversion associated with endometrial polyps. *Obstetrics & Gynecology*.

[11] Beringer R. M., Patteril M. (2004). Puerperal uterine inversion and shock. *The British Journal of Anaesthesia*.

[12] Eckhart J., Poulose T., Fox R. (2006). Occult acute uterine inversion. *The Journal of Obstetrics and Gynaecology*.

[13] Momin A. A., Saifi S. G. A., Pethani N. R., Mitha S. H. (2009). Sonography of postpartum uterine inversion from acute to chronic stage. *Journal of Clinical Ultrasound*.

[14] Lewin J. S., Bryan P. J. (1989). MR imaging of uterine inversion. *Journal of Computer Assisted Tomography*.

[15] Salomon C. G., Patel S. K. (1990). Computed tomography of chronic nonpuerperal uterine inversion. *Journal of Computer Assisted Tomography*.

[16] Duri D., Cugini U., Olivuzzi M., Del Frate G. (2008). Acute postpartum uterine inversion: report of two cases. *International Journal of Obstetric Anesthesia*.

[17] Achanna S., Mohamed Z., Krishnan M. (2006). Puerperal uterine inversion: a report of four cases. *The Journal of Obstetrics and Gynaecology Research*.

[18] O’Sullivan J. V. (1945). Acute inversion of the uterus. *BMJ*.

[19] Majumdar A., Saleh S., Bird A., Kumarage I. (2010). Successful conservative management of inversion of a fibroid uterus by hydrostatic balloon. *Journal of Obstetrics and Gynaecology*.

[20] Majd H. S., Pilsniak A., Reginald P. W. (2009). Recurrent uterine inversion: a novel treatment approach using SOS Bakri balloon. *BJOG: An International Journal of Obstetrics and Gynaecology*.

[21] Haultain F. (1901). The treatment of chronic uterine inversion by uterine hysterotomy. *BMJ*.

[22] Huntington J. L., Irving F. C., Kellog F. S. (1928). Abdominal reposition in acute inversion of the puerperal uterus. *American Journal Obstetrics and Gynecology*.

[23] Spinelli P. G. (1897). Inversione uterina. *Rivista di Ginecologia Contemporanea*.

[24] John R. P. (1958). Uterine inversion treated with the Kustner-Lede operation & pregnancy 10 years later. *Prensa Medical Argentina*.

[25] Garrett-Albaugh S., Stitely M. L., Millan L., Hochberg C. (2011). Chronic postpartum uterine inversion treated by abdominal replacement and cerclage. *The West Virginia Medical Journal*.

[26] Shepherd L. J., Shenassa H., Singh S. S. (2010). Laparoscopic management of uterine inversion. *Journal of Minimally Invasive Gynecology*.

[27] Auber M., Darwish B., Lefebure A., Ness J., Roman H. (2011). Management of nonpuerperal uterine inversion using a combined laparoscopic and vaginal approach. *American Journal of Obstetrics and Gynecology*.

